# EXAMINING VETERANS’ CHOICES OF HIGH-QUALITY MEDICARE SKILLED HOME HEALTH CARE AGENCIES

**DOI:** 10.1093/geroni/igad104.2288

**Published:** 2023-12-21

**Authors:** Katherine Kennedy, Frank DeVone, Emily Corneau, Portia Cornell, Pedro Gozalo, Kate Magid, Christine Jones, Kali Thomas

**Affiliations:** Providence VA Medical Center, Providence, Rhode Island, United States; Department of Veteran Affairs, Providence, Rhode Island, United States; Providence VAMC, Providence, Rhode Island, United States; Providence VA Medical Center, Providence, Rhode Island, United States; Brown University, School of Public Health, Providence, Rhode Island, United States; Veterans Health Administration, Aurora, Colorado, United States; University of Colorado, Centennial, Colorado, United States; Johns Hopkins University, Baltimore, Maryland, United States

## Abstract

Medicare-certified skilled home health helps Veterans restore function post-hospitalization. Our objective was to understand the quality of VA contracted home health agencies (HHAs) available to Veterans (i.e., had at least 1 VA-paid episode of care in the 12 months) relative to the local market. For comparison, about one-third (30.4%) of traditional Medicare beneficiaries received care from high-quality HHAs (4- or 5-stars on quality of care) in 2015 (Schwartz et al., 2019). We used the Home Health CAHPS (Consumer Assessment of Healthcare Providers and Systems) 2016-2019 quarterly patient experience of care overall ratings (possible values: 1-5) to define a high-quality agency as those with 4- or 5-star ratings. We matched the quarterly ratings with the discharge date from a VA Medical Center to create HHA choice sets per eligible Veteran discharge. A choice set contains all contracted and non-contracted HHAs that serve a Veteran’s Zip Code. Among HHAs proximal to Veterans’ homes across all years (N= 10,507 unique HHAs), 54.8% were high-quality (n=12,449 HHA-years), 30.1% were 3-star quality (n=6,835 HHA-years), and 15.1% were low-quality (1- or 2- stars) (n=3,444 HHA-years). Among discharges, 53.2% (n=28,081) of the discharges went to high-quality HHAs and 15.7% (n=8,294) went to low-quality HHAs. Veterans tended to live in areas with access to high-quality home health care, and most discharges mapped to high-quality HHAs. However, there is room to improve Veterans’ access to high-quality skilled home health and more to understand about VA contracting practices and the HHA selection process.

